# CasRx-mediated RNA targeting prevents choroidal neovascularization in a mouse model of age-related macular degeneration

**DOI:** 10.1093/nsr/nwaa033

**Published:** 2020-03-03

**Authors:** Changyang Zhou, Xinde Hu, Cheng Tang, Wenjia Liu, Shaoran Wang, Yingsi Zhou, Qimeng Zhao, Qiyu Bo, Linyu Shi, Xiaodong Sun, Haibo Zhou, Hui Yang

**Affiliations:** 1 Institute of Neuroscience, State Key Laboratory of Neuroscience, Key Laboratory of Primate Neurobiology, CAS Center for Excellence in Brain Science and Intelligence Technology, Shanghai Research Center for Brain Science and Brain-Inspired Intelligence, Shanghai Institutes for Biological Sciences, Chinese Academy of Sciences, China; 2 College of Life Sciences, University of Chinese Academy of Sciences, China; 3 Department of Ophthalmology, Shanghai General Hospital (Shanghai First People's Hospital), Shanghai Jiao Tong University School of Medicine, Shanghai Key Laboratory of Fundus Diseases, China; 4 Hui-Gene Therapeutics Co., Ltd. China

## Abstract

RNA-targeting CRISPR system Cas13 offers an efficient approach for manipulating RNA transcripts in vitro. In this perspective, we provide a proof-of-concept demonstration that Cas13-mediated Vegfa knockdown in vivo could prevent the development of laser-induced CNV in mouse model of Age-related macular degeneration.

Age-related macular degeneration (AMD), characterized by the development of choroidal neovascularization (CNV), is a leading cause of vision deterioration in adults over the age of 50 [1]. An angiogenic growth factor vascular endothelial growth factor A (VEGFA) plays a crucial role in CNV pathogenesis and anti-VEGFA therapy using humanized antibodies has been widely used in treating AMD, with the therapeutic effects maintained by regular injections of antibodies [2,3]. Two recent studies showed in a mouse model of AMD that permanent *Vegfa* gene disruption could be induced by spCas9 or LbCpf1 editing [4,5]. However, risks associated with permanent DNA modifications, including unwanted off-target and on-target effects, need to be considered [6,7].

The Cas13 protein family was recently shown to be a programmable RNA-targeting CRISPR system [8–14], which could mediate RNA knock-down with high efficiency and specificity relative to other existing RNA-interference approaches [8,9,12]. Several Cas13 proteins have been identified, among which CasRx (also named RfxCas13d) has the smallest size and highest RNase activity [12,13]. Here, we examine the potential application of the CasRx system for *in vivo* gene therapy, using a laser-induced mouse model of AMD. Our results show that adeno-associated viral (AAV)-delivered CasRx could knock down *Vegfa* transcripts efficiently, resulting in a significant reduction in the CNV area in this AMD model.

We first identified two CasRx targeting sites that are conserved in the human and mouse *Vegfa* gene. To achieve efficient *Vegfa* mRNA knock-down, two guide RNAs (gRNAs) targeting these two sites respectively were designed (Fig. 1a). We found that the transient transfection of vectors expressing CasRx and the gRNA resulted in a marked reduction in the *Vegfa* mRNA level in cultured human 293T cells (12% ± 3.5%, s.e.m.) and mouse N2a cells (29.5% ± 8.4%, s.e.m.) within 2 days, as compared with cells transfected with the control vector (Fig. 1b and c). The VEGFA protein levels were also significantly reduced in mouse N2a cells (Fig. 1d). To determine the targeting specificity of CasRx, we performed a transcriptome-wide RNA-seq analysis. Besides *Vegfa*, the expression levels of many other genes were changed and more than half of the top-ranked genes with altered expression were related to *Vegfa* according to previous studies (Supplementary Fig. 1 and Supplementary Table 1). To investigate the knock-down efficiency of CasRx in the normal mouse retina, we intravitreally injected AAVs encoding CasRx and a dual-gRNA array targeting *Vegfa* (referred to as AAV-CasRx-*Vegfa*). Three weeks after injection, the choroid–retinal pigment epithelial tissue complex was isolated for qPCR analysis (Fig. 1e and f). We observed the expression of AAV-CasRx-*Vegfa* (Fig. 1g) and found that *Vegfa* transcripts in the treated eye were potently suppressed, as compared with those in the contralateral eye injected with PBS (Fig. 1h).

**Figure 1.**
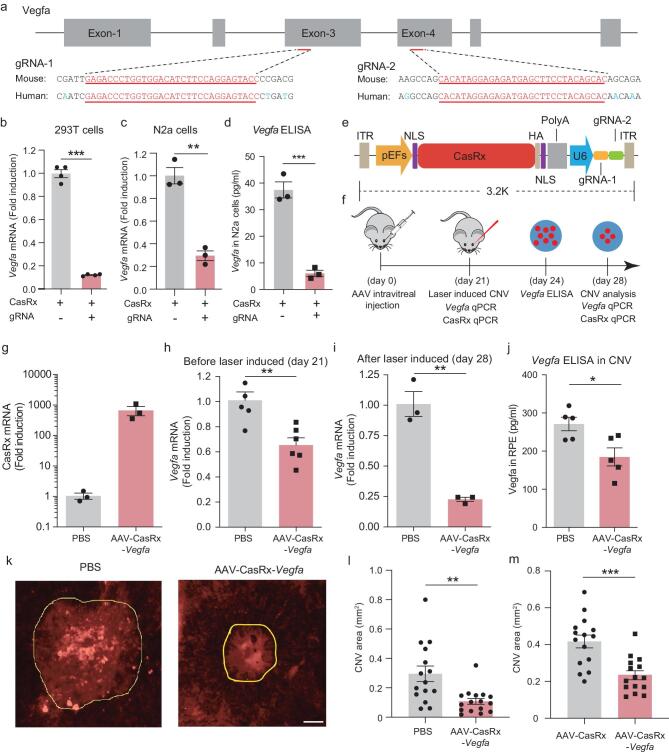
AAV-mediated delivery of CasRx reduces the area of CNV in a mouse model of AMD. (a) Schematic illustration of the targeting sites. The CasRx targeting sites are conserved in the human and mouse *Vegfa* gene. (b) and (c) Transient transfection of AAV vectors can potently knock down *Vegfa* in both human 293T cells (*n* = 4 repeats, *P* < 0.0001, *t* = 25.02) and mouse N2a cells (*n* = 3 repeats, *P* = 0.0011, *t* = 8.425). (d) VEGFA protein levels (*n* = 3 repeats, *P* < 0.01, t = 9.675). (e) Schematic showing AAV-CasRx-*Vegfa.* (f) Schematic of the experimental procedure. AAV-CasRx-*Vegfa* was intravitreally injected into one eye and AAV-CasRx was injected into the other eye as a control, 21 days before laser burn. Three weeks after AAV infection, the transcription level of *Vegfa* mRNA was analyzed without laser burn. VEGFA protein levels were quantified by ELISA 3 days after laser burn. CasRx and *Vegfa* mRNA levels as well as the area of CNV were measured 7 days after laser burn. (g) CasRx mRNA levels without laser burn, 21 days after AAV injection (*n* = 3 mice). (h) and (i) *Vegfa* mRNA levels before or 7 days after laser burn (before laser burn: *n* = 6 mice, *P* = 0.002, *t* = 4.059; after laser burn: *n* = 3 mice, *P* = 0.002, *t* = 7.583). (j) VEGFA protein levels 3 days after CNV induction (*n* = 5 mice, *P* = 0.019, *t* = 2.928). (k) Representative CNV images injected with the PBS or AAV-CasRx-*Vegfa*, 7 days after laser burn. The area of CNV is indicated by the yellow line. Scale bar: 200 μm. (l) and (m) The CNV area. A data point represents a laser burn and in total four laser burns were induced in each eye (PBS + AAV-CasRx-*Vegfa*: *n* = 4 mice, *P* = 0.002, *t* = 3.39; AAV-CasRx + AAV-CasRx-*Vegfa*: *n* = 4 mice, *P* = 0.0002, *t* = 4.292). All values are presented as mean ± s.e.m. *P < 0.05; **P < 0.01; ***P < 0.001, unpaired *t*-test.

We next created the AMD mice by inducing CNV in both eyes by laser irradiation (Supplementary Fig. 2a and b; also see the ‘Methods’ section). To investigate the potential usefulness of an mRNA knock-down approach for treating AMD, we injected AAV-CasRx-*Vegfa* into one eye of the mouse and PBS into the other eye as control (Fig. 1f). Induction of CNV was performed in both eyes 3 weeks later. After laser burn, we confirmed successful infection of AAV-CasRx-*Vegfa* (Supplementary Fig. 3a). Furthermore, we found that the levels of *Vegfa* mRNA and VEGFA protein were significantly lower in the AAV-injected eye as compared with those in the contralateral PBS-injected eye (mRNA, 22.7% ± 1.8% s.e.m., *P* = 0.002; protein, 68.2% ± 8.7%, s.e.m., *P* = 0.019; unpaired *t*-test) (Fig. 1i and j). Thus, intravitreal injection of the *Vegfa* mRNA-targeting AAV was efficient to knock down VEGFA expression in the injected eye. The therapeutic effect of this CasRx approach was assessed by quantifying the CNV area 7 days after laser treatment. Our results showed that *Vegfa*-targeting AAV markedly reduced the area of CNV at two different levels of laser irradiation, as compared with the control eyes injected with PBS (Fig. 1k and l, and Supplementary Figs 3b, 4a and b; 180 mW, 66% ± 7.8%, s.e.m., *n* = 6 mice, *P* = 0.004; 240 mW, 36.5% ± 6.9%, s.e.m., *n* = 4 mice, *P* = 0.002; unpaired *t*-test). Reduction of CNV was also confirmed by injecting AAV-CasRx-*Vegfa* into one eye and AAV-CasRx with no gRNA into the other eye as control (Fig. 1m and Supplementary Fig. 4c). To evaluate the potential toxicity of AAV-CasRx-*Vegfa*-mediated gene knock-down, we performed electroretinography (ERG) recording in mice at 1 and 2 months after the subretinal injection. Our results showed that there is no significant change in the responses in mice injected with AAV-CasRx-*Vegfa* compared to that in mice injected with PBS (Supplementary Fig. 5a and b). In addition, we examined the expression level of opsin in the retina at around 1 month after AAV injection. We found that injection of AAV-CasRx-*Vegfa* did not affect the opsin-positive areas (Supplementary Fig. 5c). Together, these results suggest that AAV-CasRx-mediated *Vegfa* knock-down is a safe way to treat AMD.

In summary, our results demonstrate that AAV-mediated delivery of CasRx can potently knock down *Vegfa* mRNA and suppress pathogenic CNV development in a mouse model of AMD, supporting the notion that the RNA-targeting CRISPR system could be useful for therapeutic purposes. The small size of CasRx is suitable for packaging with multiple gRNAs in a single AAV vector for *in vivo* delivery. Notably, AAV-delivered CasRx has the potential for sustained corrective effects on protein expression for up to 2 years with a single injection [15]. The risks associated with mRNA editing could be lower than that of DNA editing, because of the existence of large number of transcripts, many of which may maintain normal functions. Thus, a CasRx knock-down approach could complement existing therapeutic strategies such as monoclonal antibodies, antisense oligonucleotides and DNA nuclease editing. Intriguingly, a recent study demonstrated that Cas13 showed potent activity against RNA viruses [16]. In the future, it is promising to examine whether CasRx could be used to inhibit the reproduction of recently emerged deadly RNA viruses such as 2019-nCoV, Ebola, MERS and Zika.

## Supplementary Material

nwaa033_Supplemental_File
